# Applying Fuzzy Fault Tree Method to Evaluate the Reliability of College Classroom Teaching

**DOI:** 10.3389/fpsyg.2021.593068

**Published:** 2021-09-14

**Authors:** Liangliang Wang, Mingfang Fan, Feng Zhang

**Affiliations:** ^1^School of Marxism, Northwestern Polytechnical University, Xi'an, China; ^2^School of Marxism, Xi'an Shiyou University, Xi'an, China; ^3^School of Marxism, Northwestern Polytechnical University, Xi'an, China; ^4^School of Mechanics, Civil Engineering and Architecture, Northwestern Polytechnical University, Xi'an, China

**Keywords:** college classroom, teaching evaluation, fuzzy fault tree, double-layer Monte Carlo, classroom teaching failure

## Abstract

The evaluation of classroom teaching quality is closely related to the development of higher education as a scientific and effective evaluation system that can provide a solid foundation for formulating educational policies. Therefore, an evaluation model of classroom teaching quality in colleges and universities is established based on the fuzzy fault tree theory with “classroom teaching failure” as the top event to effectively evaluate the reliability of college classroom teaching and optimize the teaching strategies. In consideration of the lack of availability and dynamics of classroom teaching data, fuzzy numbers are used to describe the probability of underlying events. In addition, the top event probability of the fuzzy fault tree is calculated by the double-layer Monte Carlo method (MCM), which analyzes the classroom teaching effect based on the fuzzy fault tree reasonable. In summary, the quantitative evaluation system of classroom teaching quality based on fuzzy fault trees can evaluate classroom teaching more comprehensively and dynamically and help to improve the teaching quality of higher education.

## Introduction

### Changes in College Classroom Teaching

The objective and effective evaluation of teaching quality is not only a key factor related to the development of students, professional development of teachers, and the quality of college education and teaching but also a basis for educational decision-making (Qiao et al., 2016), even related to the personal stress and anxiety of the undergraduates (Vilvens et al., 2021). The traditional classroom teaching evaluation methods are limited by many factors, such as technology, system, and some human factors (Lee and Mccabe, 2020) in the process of their implementation that leads to objectivity and even unfairness. The mathematical model may exclude human factors, making the evaluation more scientific, objective, and measurable (Sun, 2021). Classroom teaching can be evaluated by teacher observation form (quantitatively evaluating college classroom teaching quality), and it includes the evaluation of classroom environment, curriculum structure, implementation effect, and teaching content (Cao and Mao, 2017). Some American colleges learn about the teaching quality of teachers and the learning effect of students by the evaluation of students on classroom teaching, which not only makes the evaluation more transparent and more effective through the combination of quantitative and qualitative methods but also enlightens China to improve the learning effect of student and teaching quality of teachers by the evaluation (Dai et al., 2019).

### Apply Fault Tree Analysis (FTA) to Teaching Evaluation

Nowadays, many fields have been affected by artificial intelligence (AI) (Ren et al., 2019; Tong and Sopory, 2019; Rieder et al., 2020; Vu and Lim, 2021), especially for the student-centered learning areas (Tan, 2020) and learning system (Banja, 2020; Li et al., 2020). The development of this kind of technology results in the methods of classroom teaching quality evaluation are diversified and operational, like neural network evaluation theory (Song et al., 2017), and structural equation construction (Chen, 2020). College classroom teaching is deemed as a complex system for a long time, which is not only related to the explicit educational factors, such as teaching content, teaching methods, and teaching means but also affected by such potential educational factors, such as the concentration of the students, learning consciousness, and learning initiative, and even teaching culture (Lu and Valaec, 2020). Also, the multimedia teaching system is becoming an important factor in the evaluation of teaching quality (Cheng et al., 2015). All the system engineering methods may be used to evaluate and analyze the teaching quality (Wen et al., 2019). In 1962, the Bell Telephone Laboratory proposed FTA technology that adopted a logical method for data analysis and this analysis technology become more and more popular in the engineering field, which is characterized by its simplicity, intuitiveness, systematicness, accuracy, predictability, and logicality (Sihombing and Torbol, 2018). In short, a fault tree, as an intuitive method of graphic visualization, can not only integrate possible causes of various faults but also helps to find the potential risks of the system, thereby predicting the fault of the system. Based on qualitative analysis, a fault tree can quantitatively calculate the failure probability of a complex system (Zhang et al., 2019; MacLeod et al., 2020) that may bring a big change for the teaching evaluation.

### Benefits of Fuzzy Fault Tree Method Application

Today, the analysis method based on the fault tree is one of the most commonly used methods in the field of reliability analysis and risk assessment (Song and Schnieder, 2018; Hu et al., 2020; Yan et al., 2021). The traditional fault tree mainly analyzes the causes of system failure and calculates the probability of the top event caused by each bottom event (Barlow and Proschan, 1975; Reay and Andrews, 2002; Wu, 2021). The evaluation method of teaching quality based on fault tree can help teachers find out the reasons why the teaching goal is not realized completely. Besides, the weak links in the process of predicting and diagnosing the faults can be found out, so that the corresponding improvement measures are taken to realize the optimization of teaching quality. The failure of classroom teaching is the main problem to be solved, and it is the top event of the fault tree. To count the occurrence probability of the top event, the first thing is to calculate the bottom event occurrence probability by the traditional analysis, which does not apply to the analysis of classroom teaching failure. Moreover, the evaluation of classroom teaching quality involves dynamic analysis, by which bottom event probability is not obtained. In addition, the complex system itself makes it impossible to describe the relationship between its components by using the traditional FTA.

For these reasons, the fuzzy number is used to describe the bottom event occurrence probability based on the traditional fault tree, and a new fuzzy fault tree model for classroom teaching is constructed. The double-layer Monte Carlo method (MCM) is used to count the top event occurrence probability of the model, realizing the effective and quantitative reliability evaluation of college classroom teaching (Baumgärtner and Binder, 1984; Barel and Vandewalle, 2019, 2021). As a result, the fuzzy fault tree method is used to figure out the factors that influence the classroom teaching effect, quantitatively evaluating the college classroom teaching quality that made the analysis more reasonable and more feasible.

## FTA Based on Fuzzy Model

### Theoretical Basis of Fault Tree

Fault tree analysis refers to a process, which starts from a possible “event,” looking for the direct and indirect causes leading to the occurrence of the top event layer-by-layer from top to bottom. When tracing back to the basic events, it uses a logical diagram to show the logical relations of these events. The fault tree has two elements, one is the event and the other is the logic gate. In the process of establishing a fault tree, the occurrence of the top event, intermediate event, and the bottom event is replaced by “event,” and logical relations between each system and part are displayed by “logic gate” (Hu et al., 2020).

The mathematical description of fault tree structure-function is as follows:


(1) $$\begin{array}{l} \Phi =\Phi \left(\bar{X}\right),\bar{X}=\left(x_{1},x_{2},\cdot \cdot \cdot ,x_{n}\right) \end{array}$$

Classic fault tree logic gates can be divided into two types: and-gate and or-gate. Based on the system functional logic, a system and its components are connected by logic gates to form a system FTA model, including input and output functions.

The structure-function of a fault tree under and-gate is:


(2) $$\begin{array}{l} \Phi \left(\bar{X}\right)=\overset{n}{\underset{i=1}{\prod }}x_{i} \end{array}$$

The structure-function of a fault tree under or-gate is:


(3) $$\begin{array}{l} \Phi \left(\bar{X}\right)=1-\overset{n}{\underset{i=1}{\prod }}\left(1-x_{i}\right) \end{array}$$

*n* is the number of bottom events, and *x*_*i*_(*i* = 1,2,…,*n*) is the bottom event.

### Fuzzy Fault Tree

The FTA of teaching quality evaluation can help the insight of teachers into the failure of achieving teaching objectives. In addition, it can find out the weak links in the teaching process by failure prediction and diagnosis, so that the countermeasures can be taken to improve the teaching quality during the teaching process, realizing the optimization of teaching quality. Since a fault tree is a logic diagram composed of specific logic gates, it can establish a tree model and analyze qualitatively and quantitatively with the help of a computer program. FTA can not only find out the causes that influence the effect of classroom teaching but also find the ways to benefit the effect of classroom teaching in improving the effectiveness of classroom teaching and comprehensive abilities of the students.

In the research results published in 1965, Professor Zadeh, an American cybernetic expert, thought that “membership function” could be used to describe the intermediate transition in phenomenon difference, which made a breakthrough in the absolute relationship between belonging and not belonging in a group of classical set theory. This important contribution of Zadeh marks the birth of fuzzy mathematics (Shi et al., 2018). Later, fuzzy mathematics and fault tree are effectively combined, and the fuzzy FTA method comes into being (Hu et al., 2019). The traditional FTA is based on an assumption, that is, the system and its components are required to have only two states: normal or failure. The existing theory requires that the occurrence probability of the top event and the basic event of the fault tree is an accurate value, which is difficult to achieve in practice. Due to the influence of various external complex factors and equipment contingency, the failure probability of each basic event is uncertain, and it is fuzzy. The mean value of the event occurrence and its confidence interval are obtained by experience and judgment. In addition, the fuzzy number can be used to describe the occurrence of the event by carrying out an effective reliability analysis.

#### Fuzzy Set and Membership Function

The extension of conception can be expressed mathematically by the conception of “set.” However, many conceptions involved in daily life are often accompanied by connotative “fuzziness,” which leads to the “ambiguity” of extension, while “classic set” requires that its result must be “clear,” that is, in terms of set A and a specific object, only one of the two is true. This indicates that the extension of fuzzy conception cannot be described by a classical set and that the conception of Zadeh's fuzzy set is more accurate in practice.

Set *U* as a domain, the so-called fuzzy set *Ã* on *U* refers to ∀*x* ∈ *U*, *x* often belongs to *Ã* to some extent of μ(μ ∈ [0, 1]), rather than *x* ∈ *Ã* or *x* ∉ *Ã*. μ is called a membership function on *U*.

#### Fuzzy Number

Set *Ã* as a normal fuzzy set in the real number field *R*. When ∀λ ∈ [0, 1], *A*_λ_ are all closed intervals. Then, *A*_λ_ = [*a*_λ_, *b*_λ_] is obtained. *Ã* is a fuzzy real number, a fuzzy number for short. All fuzzy numbers are recorded as $\tilde{R}$.

#### Cut Set

If *Ã* ∈ ζ(*X*), when λ ∈ [0, 1], then


(4) $$\begin{array}{l} A_{\lambda }=\left\{x\in X|Ã\left(x\right)\geq \lambda \right\} \end{array}$$

*A*_λ_ is λ -cut set of fuzzy set *Ã*, or called λ -level set of *Ã*.

And


(5) $$\begin{array}{l} A_{\bar{\lambda }}=\left\{x\in X|Ã\left(x\right)>\lambda \right\} \end{array}$$

*A*_λ_ is strong λ -cut set of fuzzy set *Ã*, or called strong λ -level set of *Ã*; λ is the threshold value or belief level.

*A*_λ_ is a classical set, rather than a fuzzy set. Because the boundary of each set is fuzzy, different belief levels [λ(0 ≤ λ ≤ 1)] should be used to determine its membership and relationship if the fuzzy conception is to be transformed. λ -cut set is a method that can transform a fuzzy set into the classical set.

#### Convex Fuzzy Set

Set *R* as a real field. And if *Ã* ∈ ζ(*R*), ∀*x*_1_, *x*_2_, *x*_3_ ∈ *R*, and *x*_1_ > *x*_2_ > *x*_3_, the following equation is obtained:


(6) $$\begin{array}{l} Ã\left(x_{2}\right)\geq Ã\left(x_{1}\right)\wedge Ã\left(x_{3}\right) \end{array}$$

Then *Ã* is a convex fuzzy set; if its height is 1, then it is a positive fuzzy set.

## Establishment of Fault Tree Regarding “Classroom Teaching Failure”

First, setting up a top event. “Classroom Teaching Failure” is taken as the top event, and the top event is analyzed layer by layer to find the causes that influence the effect of college classroom teaching. Thus, the fault tree of college classroom teaching is established.

Second, building intermediate events. The factors affecting the occurrence of classroom teaching events include not only teachers and students, but also the potential teaching environment. These three factors are regarded as composition conditions in the classroom teaching process and considered as the intermediate event of the fault tree.

Third, defining basic events. Take the factors that may affect the top event as the basic events of the fault tree. Because of the complexity of the classroom teaching environment, 10 events are involved. As for teachers, the factors concerned are teaching method, teaching speed, and charm of a teacher; in terms of students, factors concerned are learning attitude, learning motivation, and learning method; concerning teaching environment, they include teaching content, class management, exercise class, and experiment class.

Fourth, representing events. The symbols of the fault tree are used to represent these events, and the events of different levels are connected into an inverted tree with appropriate logic gates so that the logical relationship between the events of the classroom teaching effect can be described.

Classroom teaching includes intermediate events, and the lack of any party cannot make classroom teaching fully reflected. The interaction between basic events, namely, intermediate events, affects the classroom learning effect. Therefore, the three levels of events should be interconnected, and gate structure is selected, in which the top event, intermediate event, and basic event are all reflected as “•.”

**Figure 1:**
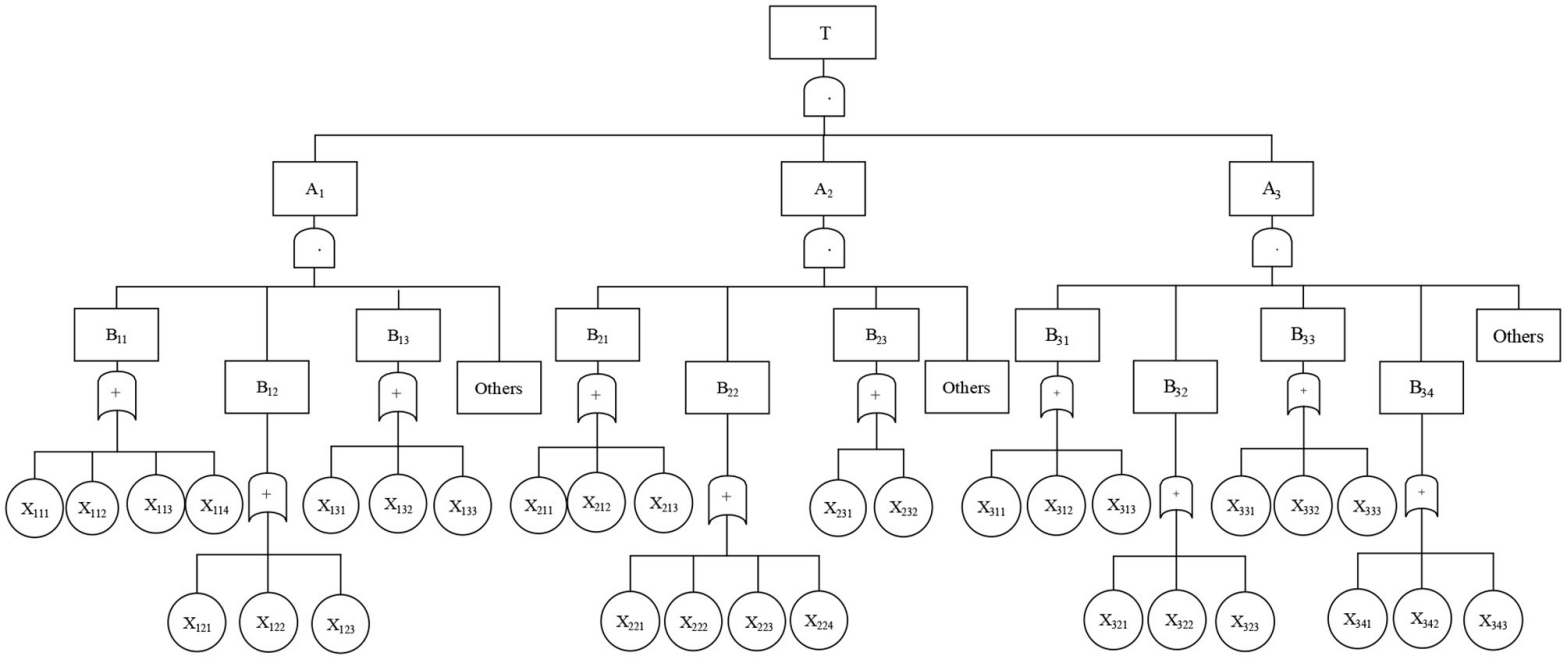
Fault tree of classroom teaching effect.

When gate structure connects the bottom events, it is expressed as “+.” The reason is that the simplification of basic events makes it easier for students to answer mutually exclusive questions. After calculation, a complete fault tree diagram of “college Classroom Teaching Failure” is displayed (Lu and Valaec, 2020), as shown in Figure 1.

In Figure 1, T is a classroom teaching failure; A_1_ is the teacher-related intermediate event, A_2_ is the student-related intermediate event, and A_3_ is the environment-related intermediate event; B_11_ is teaching method, B_12_ is personal charm, and B_13_ is teaching progress; B_21_ is learning attitude, B_22_ is learning motivation, and B_23_ is learning method; B_31_ is class exercises, B_32_ is class experiments, B_33_ is teaching content, and B_34_ is classroom management; X_111_ is proficiency, X_112_ is vision, X_113_ is theory to practice, and X_114_ is suitable teaching; X_121_ is classroom atmosphere, X_122_ is teacher model, and X_123_ is teaching language; X_131_ is fast pace, X_132_ is normal pace, and X_133_ is slow pace; X_211_ is attention, X_212_ is homework completion, and X_213_ is tutoring time; X_221_ is subsequent courses, X_222_ is interests and hobbies, X_223_ is trouble on exam, and X_224_ postgraduate preparation needs; X_231_ is the use of preview and review and X_232_ is the use of answer and question methods; X_311_ is necessary, X_312_ is unnecessary, and X_313_ is indifferent; X_321_ is good effect, X_322_ is general effect, and X_323_ is normal; X_331_ is more than 80% relevant, X_332_ is more than 60% relevant, and X_333_ is less than 60% relevant; X_341_ is strict, X_342_ is appropriate, and X_343_ is loose.

The factors that cause classroom teaching failure mainly include teachers, students, and the environment, which are regarded as the intermediate events of the fault tree. The bottom events are determined from the three aspects of teachers, students, and environments, that is, the factors that affect the top events as the basic event of the fault tree. They mainly include teaching methods of the teachers, charm of the teachers, and their teaching speed; attitudes of students toward study, their motivation for study, and their learning methods; exercises, experiments, teaching content, and classroom management. Based on the newly established fault tree model and the existing relevant information (Wen et al., 2019; Lu and Valaec, 2020), the fuzzy number of occurrence probability of each bottom event is finally determined by combining the experiences of different experts following fuzzy mathematics theory, as shown in Table 1.

**Table 1:** Event occurrence probability of classroom teaching failure.

**Label**	**An intermediate value of failure probability**	**Left utility value**	**Right utility value**
X_111_	0.69	0.685	0.695
X_112_	0.67	0.665	0.675
X_113_	0.57	0.565	0.575
X_114_	0.64	0.635	0.645
X_121_	0.65	0.645	0.655
X_122_	0.70	0.695	0.705
X_123_	0.76	0.755	0.765
X_131_	0.59	0.585	0.595
X_132_	0.38	0.375	0.385
X_133_	0.03	0.025	0.035
X_211_	0.73	0.725	0.735
X_212_	0.72	0.715	0.725
X_213_	0.76	0.755	0.765
X_221_	0.43	0.425	0.435
X_222_	0.17	0.165	0.175
X_223_	0.33	0.325	0.335
X_224_	0.07	0.065	0.075
X_231_	0.80	0.795	0.805
X_232_	0.88	0.875	0.885
X_311_	0.88	0.875	0.885
X_312_	0.05	0.045	0.055
X_313_	0.07	0.065	0.075
X_321_	0.35	0.345	0.355
X_322_	0.50	0.495	0.505
X_323_	0.15	0.145	0.155
X_331_	0.40	0.395	0.405
X_332_	0.90	0.895	0.905
X_333_	0.10	0.095	0.105
X_341_	0.63	0.625	0.635
X_342_	0.27	0.265	0.275
X_343_	0.10	0.095	0.105

## Probability Analysis of Classroom Teaching Failure Under the Fuzzy Model

The theoretical basis of the MCM is the law of large numbers of probability theory and Bernoulli's theorem. Indeed, it is a kind of statistical sampling test or random simulation calculation method that can be used for relevant random variables. By estimating and describing the statistics of a function, the numerical calculation method of approximate solutions in engineering technology problems is adopted (Chong and Hu, 2020). The double-layer MCM is used for sampling (Zhang et al., 2013; Shen et al., 2019), and then the simulation method is used to solve the probability of failure of the top event.

The failure transfer probability function can be obtained by analyzing the fault tree structure of the classroom teaching effect. After the membership function of the bottom, the event is determined, the upper event occurrence probability of each logic gate in the fault tree can be solved according to the system fault tree under the fuzzy model (Chen, 2018), and then the influence degree of classroom teaching effectiveness can be obtained, as shown in Table 2 below.

**Table 2:** Event probability of each layer.

**No**.	**Probability of failure**	**No**.	**Probability of failure**	**No**.	**Probability of failure**
		T	[0.2091, 0.2440]		
A_1_	[0.7151, 0.7304]	A_2_	[0.6661, 0.6853]	A_3_	[0.4507, 0.4759]
B_11_ B_21_ B_31_ B_34_	[0.9832, 0.9850] [0.9808, 0.9829] [0.8884, 0.8995] [0.7506, 0.7632]	B_12_ B_22_ B_32_	[0.9735, 0.9761] [0.6970, 0.7133] [0.7172, 0.7302]	B_13_ B_23_ B_33_	[0.7471, 0.7596] [0.9744, 0.9776] [0.9425, 0.9494]

Since the convergence criterion of the MCM is used, the number of outer layer sampling is set as 800, the number of inner layer sampling as 8,000, and the probability interval value of “teaching failure” of top event calculated by double-layer MCM is [0.2091, 0.2440].

**Figure 2:**
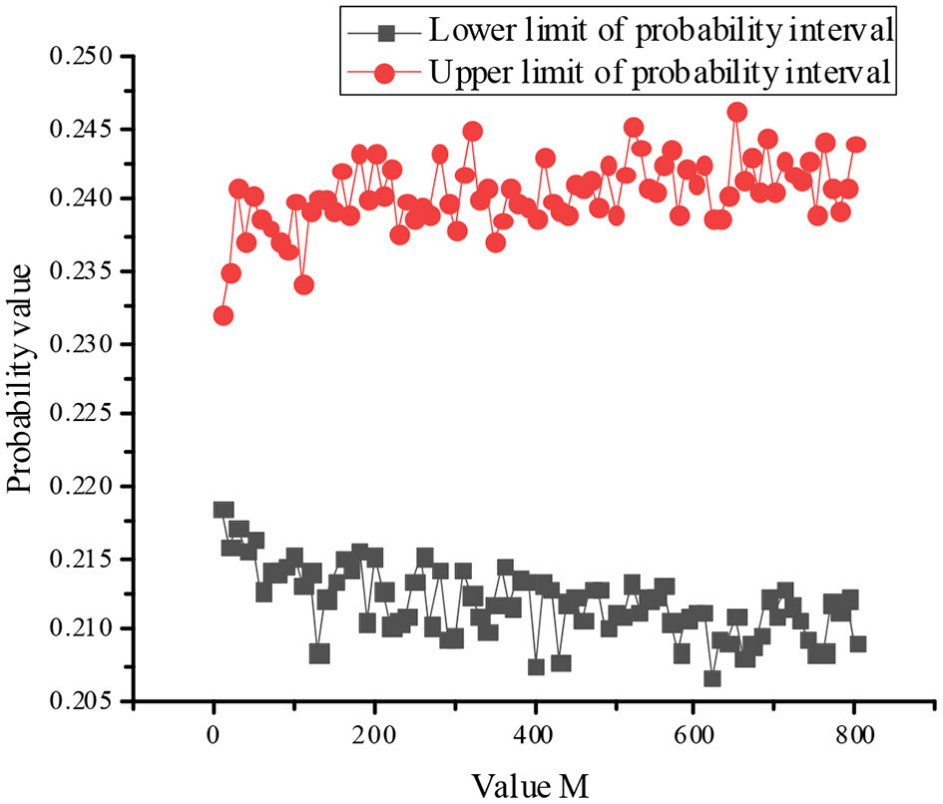
Change law of failure probability with number change of outer layer sampling.

The influence of value M on the interval convergence is shown in Figure 2 as follow.

**Figure 3:**
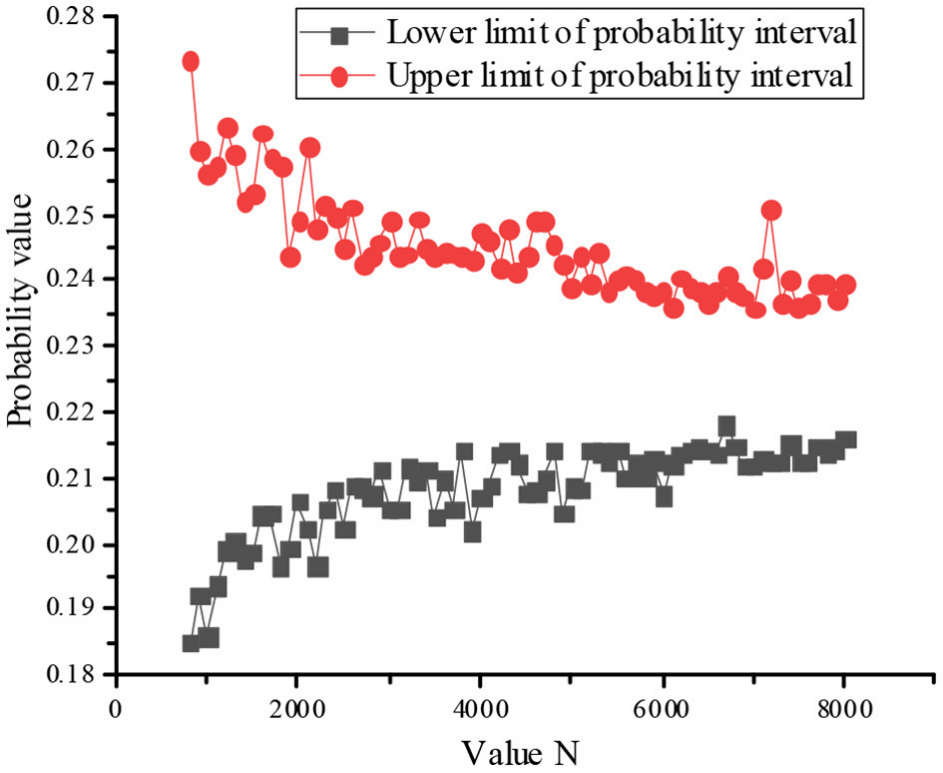
Change law of failure probability with number change of inner layer sampling.

The influence of value N on probability interval convergence is shown in Figure 3 as follow.

## Conclusion

The FTA method is further optimized, that is, fuzzy mathematics is introduced to describe the uncertainties of occurrence probability of the bottom event that may cause college classroom teaching failure. Then, the probability interval of teaching failure is simulated by the double-layer MCM (Chen et al., 2019). Finally, the probability interval of college classroom teaching effect is evaluated quantitatively. This study improves and innovates a traditional evaluation method for one thing, and embodies the advantages of the fuzzy FTA method for another. The probability analysis results of classroom teaching failures show that the probability of top events is [0.2091, 0.2440], which means that the students believe that the probability of classroom teaching failure is 20.91–22.4%. The representation theorem of the fuzzy set theory is used to calculate the probability of the fuzzy number of top events and the importance of bottom events. The calculation shows that the establishment of the FTA model based on fuzzy numbers provides a reference for evaluating the teaching quality of teachers in the classroom (Chen, 2019).

### Comprehensiveness

Different from the single FTA, this evaluation method fully considers the uncertainties of bottom event and intermediate event into the model, so that it can comprehensively and reasonably evaluate the reliability of college classroom teaching systems (Feng et al., 2020), which makes the factors that affect the classroom teaching failure appear and provides a variety of reference factors for colleges to formulate the teaching evaluation program. It is fundamentally helpful for the improvement of college teaching quality in China.

### Relevance

Educational researchers and educators can intuitively find the logical relationship between the factors that cause classroom teaching failure. When a cause (such as a bottom event and intermediate event) of classroom teaching failure occurs, the overall teaching system of classroom teaching may be traced back to its source, so that its direct and indirect causes are figured out to correct the teaching failure, which is beneficial for the improvement of classroom teaching effect (Feng and Chen, 2020).

### Dynamics

The evaluation method of college classroom teaching quality is highly operational and dynamic, and the fault tree method plays an important role in computer database construction. Meanwhile, it can also improve the classroom teaching quality in a dynamic evaluation system, achieving the overall education goal of colleges (Deng et al., 2021).

### Expansibility

The evaluation model has certain expansion space as well. With the change and development of the current situation of education and teaching, many non-human and pre-set factors may affect the effects of classroom teaching. For example, the COVID-19 outbreak in 2019 and other non-conventional factors may be included in the evaluation model. Thus, this model needs to be further studied and developed considering more certain and uncertain factors in the future.

## Data Availability Statement

The raw data supporting the conclusions of this article will be made available by the authors, without undue reservation.

## Ethics Statement

The studies involving human participants were reviewed and approved by Northwestern Polytechnical University Ethics Committee. The patients/participants provided their written informed consent to participate in this study. Written informed consent was obtained from the individual(s) for the publication of any potentially identifiable images or data included in this article.

## Author Contributions

LW developed the theoretical formalism, conceived of the presented idea, and wrote the manuscript with support from MF and FZ. Specially FZ fabricated the samples and performed the analytic calculations. MF supervised the project. LW and MF contributed to the final version of the manuscript. All authors listed have made a substantial, direct and intellectual contribution to the work, and approved it for publication.

## Funding

This study was supported by the Shaanxi Association of higher education 2019 Higher Education Scientific Research Project (XGH19080) and Northwestern Polytechnical University Degree and Graduate Education Research Fund Project (2019–2020).

## Conflict of Interest

The authors declare that the research was conducted in the absence of any commercial or financial relationships that could be construed as a potential conflict of interest.

## Publisher's Note

All claims expressed in this article are solely those of the authors and do not necessarily represent those of their affiliated organizations, or those of the publisher, the editors and the reviewers. Any product that may be evaluated in this article, or claim that may be made by its manufacturer, is not guaranteed or endorsed by the publisher.
